# Effort and reward imbalance factors motivating Namibian professional nurses to participate in continuous professional development: A confirmatory factor analysis

**DOI:** 10.4102/hsag.v25i0.1313

**Published:** 2020-12-21

**Authors:** Tekla S.N. Mbidi, Anneleen Damons

**Affiliations:** 1 Department of Nursing and Midwifery, Faculty of Medicine and Health Sciences, Stellenbosch University, Cape Town, South Africa; 2 International University of Management, Windhoek, Namibia

**Keywords:** effort rewards, confirmatory factor analysis, continuous professional development, Namibia, professional nurses

## Abstract

**Background:**

To improve professional development, it is important to understand the motivational factors behind nurses’ participation in specific types of continuous professional development activities. Effort–rewards imbalance (ERI) posits an imbalance between high efforts spent at work and low rewards sometimes received in turn. However, professional nurses have various ERIs that can influence their reasons to participate in continuous professional development activities.

**Aim:**

The purpose of this article was to propose a model for selected ERI factors, which motivate professional nurses to participate in continuous professional development activities.

**Setting:**

Two hundred and forty-one professional nurses working in a public national referral hospital in Namibia participated in the study.

**Methods:**

Survey data on professional nurses’ reasons and motivations to participate in the professional development activities were analysed using a literature-based framework on ERI and reasons for participation in continuous professional development. The survey data were analysed for reflective relationships of ERI and reasons for participation in continuous professional development activities. A confirmatory factor analysis method using IBM SPSS AMOS version 23 was used to develop and validate the effort–reward motives for a continuous professional development model.

**Results:**

Four effort-reward imbalance factors were derived from sixteen CPD motives. The reflective factors were (1) extrinsic efforts, (2) intrinsic efforts, (3) reward motives, and (4) over-commitment motives. The four conceptual factors made up a second-order effort-reward motives factor for the nurses’ reasons to take part in continuous professional development activities.

**Conclusions:**

The results of this study show that professional nurses consider taking part in continuous professional development activities in order to carry out their work better but not as a way to increase chances of promotion. The study also concluded that the older professional nurses tend to have higher intrinsic effrot motivation than their younger counterparts. Thus, nurses could use these findings to understand the reasons which motivate them to develop professionally.

## Introduction

Professional development is the active process enabling professional nurses to make progress in their nursing careers (Rahimaghaee, Nayeri & Mohammadi 2010). Similarly, Spear (2016) defines professional development as a process of improving and increasing nursing competencies and knowledge through access to nursing education and training opportunities in the workplace or through the observation of other nurses performing nursing tasks. Additionally, continuing professional development (CPD) of nurses is crucial to maintaining a competent and motivated workforce that can provide safe patient care (Pool et al. 2016).

There is a growing notion that nurses participate in different continuous professional development activities when they are motivated (Badu-Nyarko 2015). Pool et al. (2016) suggested that professional development is largely influenced by employees’ values, norms, attitudes and competencies. However, these values, norms and motives are influenced by their exposure to occupational stressors, such as high workloads, staff shortages, low promotion prospects, working with limited resources and sometimes by a lack of opportunities for career growth (Darboe, Lin & Kuo 2016). As such, it is important to note that high efforts and low rewards imbalance in most healthcare professionals have caused high levels of job dissatisfaction (Schulz et al. 2009). Thus far, not much is known about the effort–reward imbalance (ERI) factors, which motivate nurses to participate in CPD activities. Consequently, this article attempts to empirically measure the level of effort–reward factors amongst Namibian nurses and synthesises various studies on ERI and participation in CPD activities. This knowledge adds to the understanding of what motivates the nurses’ reasons to participate in professional development activities within an occupationally stressful environment.

## Background

Goedhart, Van Oostveen and Vermeulen (2017) noted that in professional practice, work environments play a major role in the performance of nurses and in the quality and well-being of patient care. Duffield et al. (2014) stated that recognition and incentives of nurses need to be supplementary to their skills and expertise in order to show that nurses’ services are valued and this would assist in reducing staff turnover. Whilst evidence from Liu et al. (2015) shows that lack of professional growth prohibits the development of a nursing team, and this may lead to a high turnover. Similarly, Cicolini, Comparcini and Simonetti (2014) elucidated that satisfying workplace environments for nurses are related to structural and psychological empowerment. Kluska, Laschinger and Kerr (2004) modelled an expanded empowerment model for nurses based on Kanter’s (1993) empowerment theory and Siegrist’s (1996) ERI model. Kluska et al.’s (2004) model links structural empowerment, psychological empowerment, over-commitment and ERI. The study adopts Kluska et al.’s (2004) model and links it with Brekelmans et al.’s (2015) CPD motives.

The CPD motives refer to the reasons and motivations for nurses to engage in CPD activities, which may arise from a range of different needs, including activities related to registration requirements, career opportunities, and personal and professional development (Brekelmans et al. 2015). Nurses are motivated to participate in CPD activities in conformance to their registration requirements, a desire to improve the standards of practice and gaining further qualifications to enhance promotion prospects (Bahn 2007; Brekelmans et al. 2015; Gould, Drey & Berridge 2007; Ryan 2003). The personal motivation of the nurse to engage in CPD is fundamental for professional development (Berings et al. 2007; Twaddell & Johnson 2007). In Namibia, the health professions council CPD directive of 2011 requires all registered health professionals to complete a series of accredited continuing education activities each year. In addition, health professionals can select continuous professional development activities at any level of learning that meet their particular needs and the demands of their practice environments (Health Professions Councils of Namibia 2011). Although participation in continuous professional development activities is mandatory, registered professionals are permitted to maintain their annual registration without submitting any proof of having attended CPD activities and this could be a reason why nurses may not be motivated to participate in professional growth activities.

Effort–reward imbalance is an established conceptualisation of work stress (Siegrist et al. 2014). In nursing, job stress has been associated with high levels of emotional strain and heavy workloads (Aiken et al. 2001), elevated higher risks of psychomedical health complaints, physical health symptoms and job dissatisfaction (Van Vegchel et al. 2001), and staff nurse empowerment (Kluska et al. 2004). The theoretical model of ERI maintains that lack of reciprocity between efforts spent and rewards received (i.e. ‘high cost or low gain’ conditions) in a core social role, the work role, defines a state of emotional distress, which is related to adverse health outcomes (Siegrist 1996).

**FIGURE 1:**
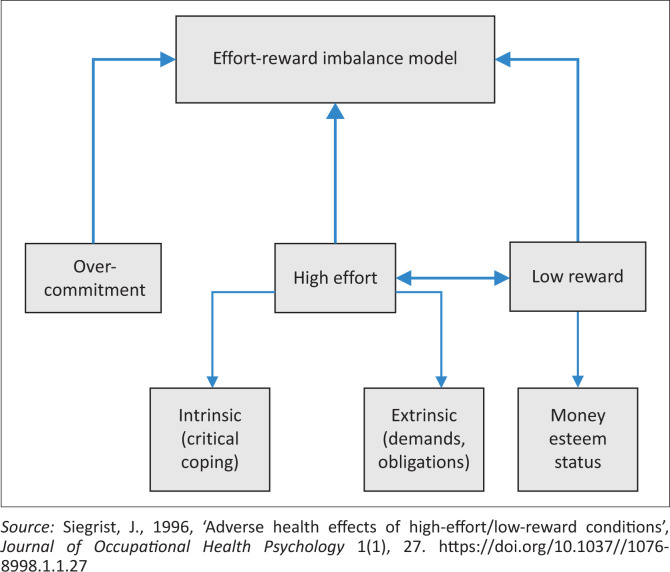
Effort–reward imbalance at work.

Siegrist et al. (2014) conceded and supported the notion of using proxy measures or partial versions of the original ERI questionnaire, which consists of 23 two-stage Likert scale items and covers the three major components of Siegrist’s model: extrinsic effort, reward and over-commitment (intrinsic effort) (Siegrist 1996). As such, this study uses proxy measures from the CPD motives to link ERI to participation in CPD activities. The study uses an extended ERI model as presented in Figure 1.

In the ERI model (Figure 1), Siegrist (1996) defined extrinsic effort as the situational job demands and obligations. Rewards for efforts spent at work consist of money, esteem and status control (including promotion prospects and job security). The intrinsic effort consists of personal coping patterns, such as the high need for control (Siegrist 1996). According to Deci and Ryan’s (2000), the self-determination theory (SDT) differences in work effort exertion may be explained by the type of work motivation employees are driven by (Dysvik & Kuvaas 2013). In SDT, intrinsic motives can be defined as the motivation to perform an activity for its own sake in order to experience the pleasure and satisfaction inherent in the activity (Deci, Connell & Ryan 1989). As such, intrinsically motivated nurses participate in CPD activities because they find them enjoyable and interesting, and that participation is its own reward.

Preckel et al. (2005) defines ‘overcommitment’ as a set of attitudes, behaviours and emotions that reflect a person’s excessive striving for approval and appreciation. The ERI model proposes that people who overcommit are exaggerating their efforts beyond levels usually considered appropriate, or that they expose themselves to high demands at work too often (Preckel et al. 2005). Consequently, these efforts diminish their potential to recover from job demands and increase their susceptibility to frustration when the expected rewards are not forthcoming (Siegrist 2001), which eventually leads to reduced participation in CPD activities.

## Aim

The purpose of this article was to propose a model for selected ERI factors, which motivate nurses to participate in continuous professional development activities.

## Research design

A quantitative correlational research design using a validated structured questionnaire survey was employed. Survey data on nurses’ reasons and motivations to participate in the professional development activities were analysed using a literature-based framework on ERI and reasons for participation in continuous professional development. The survey data were analysed for reflective relationships of ERI and reasons for participation in continuous professional development. A confirmatory factor analysis (CFA) method using IBM SPSS AMOS version 23 was applied to develop and validate the effort–reward motives for a continuous professional development model.

### Research setting and participants

The study was conducted in a public national referral and a teaching hospital in Namibia. The hospital has a bed capacity of 855 beds. Nurses make up the largest group of employees (*n* = 650) of which 292 are professional nurses, 38 are senior professional nurses, 11 professional nurses or clinical instructors, one nurse manager and 308 are enrolled nurses. In this study, 342 nurses constituted the population as the total number of professional nurses working in the health facility and who were eligible to participate in the study. In this study, the researcher did not use a sample but targeted the total population (*N* = 342). As a result, 270 questionnaires were distributed; a total of 241 questionnaires were completed and returned representing an 89% response rate. The pilot study was conducted with 34 participants who were excluded from the main study. The remaining 38 participants did not reach on the data collection days.

### Data collection instrument

Data in this study were collected using an already validated structured questionnaire, the professional development nurses’ instrument (Q-PDN). The Q-PDN was developed by Brekelmans et al. (2015) in order to measure several aspects of CPD amongst nurses in the Netherlands. The questionnaire measured four constructs, namely, CPD motives, importance attached to CPD, conditions deemed needed for CPD and actual CPD activities undertaken. The instrument had 54 items, which were rated on a five-point Likert scale. The researcher obtained permission to use the instrument and permission was also granted by the author to make necessary changes for the instrument to be fit for use in the Namibian context. The changes made to the questionnaire included aligning the demographic information with Namibian certifications, positions and education levels. Effort–reward motivation was measured from Part 2A of the questionnaire, which covered CPD motives. The following sentence is an example of a question: I take part in CPD activities in order to meet the requirements for registration in the future. Additionally, the response anchors were changed to a four-point Likert scale rating instead of the original five-point scale. The response anchors used were as follows: (1) mainly disagree, (2) partly disagree, (3) partly agree and (4) mainly agree. The instrument was reliable to use in this study with a Cronbach’s alpha (α) of 0.848.

### Data collection

Data collection was conducted after ethics approval from Stellenbsoch University Health Research Ethics Committee, as well as from the ethics committee of the health facility in which the study was conducted. Data collection for this study was conducted during the period 30 April 201731 May 2017. The pilot study and the main study were administered 6 days apart, and given the nature of the work for the professional nurses, the researcher decided to exclude pilot study participants to minimise the risk of receiving half-completed questionnaires and also avoid response bias. To ensure the anonymity of participants, they were requested to place their completed questionnaires in a provided sealed envelope for placement in a questionnaire box. The signed consent forms and completed questionnaires were then documented in a register, which was kept by the researcher. The pilot test was conducted on 10% (*n* = 34) of the professional nurses. The pilot study was conducted in the hospital where the main study was conducted because the health facility is the only public national referral hospital in the whole country and the conditions are not the same as in other hospitals in Namibia. According to Thabane et al. (2010:9), pilot study participants can be excluded from the main study in order to avoid potential response bias.

### Data analysis

#### Data screening

The study followed Gaskin’s (2017) procedure for CFA. The procedure starts with data screening to ensure that the data are useable, reliable and valid for testing causal theory. In line with Gaskin’s (2017) approach, exploratory factor analysis (EFA) was utilised to prepare the variables to be used for more efficient CFA.

The 16 CPD motive items were subjected to principal axis factor analysis (PAF) using SPSS version 23. Principal axis factoring was used instead of principal component analysis because it accounts for co-variation, whereas PCA accounts for total variance (Gaskin 2017). Prior to performing PAF, the suitability of data for factor analysis was confirmed. Inspection of the correlation matrix revealed the presence of many coefficients of 0.3 and above (communalities). The Kaiser–Meyer–Olkin value was 0.79, exceeding the recommended value of 0.6 (Tabachnick & Fidell 2007) and Bartlett’s test of sphericity reached statistical significance, supporting the factorability of the correlation matrix. Principal axis factor analysis revealed the presence of four factors with eigenvalues exceeding 1, explaining a total of 51.29% of the variance, with factor 1, factor 2, factor 3 and factor 4 contributing 24.3%, 12.0%, 7.9% and 7.0% of the variance, respectively.

To aid in the interpretation of these four factors, Varimax rotation was performed. The varimax rotation was used to simplify the interpretation of the factors by focusing on their shared variance, as well as limit their location within the factor space. This resulted in the smallest number of variables that can possibly load on a factor with high loadings (Kline 1994). Consequently, small coefficients of factors with loadings less than 0.30 were also suppressed. Table 1 presents the results of the principal axis factor analysis performed using SPSS version 23.

**TABLE 1:** Descriptive and reliability analysis results for effort–reward imbalance factors.

Codes	EFA factors for I take part in CPD activities:	Career		PPD		Required		Mean rank	Exploratory factor analysis (KMO = 0.79)			
		*M*	SD	*M*	SD	*M*	SD		Loadings	Communalities	Eigenvalues	Cronbach’s alpha
**Factor 1**	**Extrinsic effort**										**3.89**	**0.67**
Part 2A2	In order that increase my chances of promotion	2.28	1.12	-	-	-	-	16	0.60	0.41	**-**	**-**
Part 2A1	In order to meet the requirements for registration in the future	-	-	-	-	2.64	1.19	15	0.51	0.31	**-**	**-**
Part 2A11	To prove to my employer that I am professionally competent	-	-	-	-	2.78	1.05	13	0.72	0.57	**-**	**-**
**Factor 2**	**Intrinsic effort**										**1.12**	**0.60**
Part 2A6	Because I consider it is important to increase the status of my profession	-	-	-	-	3.46	0.69	6	0.48	0.46	**-**	**-**
Part 2A5	To improve my current qualifications	-	-	3.10	0.98	-	-	11	0.48	0.41	**-**	**-**
Part 2A4	To increase my professional status	3.42	0.74	-	-	-	-	8	0.62	0.42	**-**	**-**
Part 2A3	I take part in CPD because further professional development is important to me	-	-	3.45	0.59	-	-	7	-	0.22	**-**	**-**
**Factor 3**	**Over-commitment motives**										**1.10**	**0.67**
Part 2A12	Because this is considered highly important in my professional development	-	-	-	-	3.49	0.68	5	0.62	0.44	**-**	**-**
Part 2A13	In order to achieve a higher level of training	3.39	0.73	-	-	-	-	9	0.47	0.39	**-**	**-**
Part 2A10	In order to increase the quality of the healthcare	-	-	3.64	0.60	-	-	2	0.36	0.36	**-**	**-**
Part 2A16	To improve my leadership abilities	2.68	1.12	-	-	-	-	14	0.49	0.29	**-**	**-**
Part 2A15	To support my career potential or choice	3.07	0.78	-	-	-	-	12	0.57	0.53	**-**	**-**
Part 2A14	I take part in CPD activities in order to make a positive contribution to nursing practice	-	-	3.67	0.57	-	-	1	0.53	0.35	**-**	**-**
**Factor 4**	**Reward motives**										**1.26**	**0.49**
Part 2A8	In order to carry out my work better	-	-	3.58	0.60	-	-	3	0.50	0.37	**-**	**-**
Part 2A7	To support my career	3.53	0.63	-	-	-	-	4	0.56	0.39	**-**	**-**
Part 2A9	I take part in CPD activities in order to meet the requirements of the organisation I work for	-	-	-	-	3.20	0.89	10	0.44	0.38	**-**	**-**

*Source:* Brekelmans, G., Maassen, S., Poell, R.F. & van Wijk, K., 2015, ‘The development and empirical validation of the Q-PDN: A questionnaire measuring continuing professional development of nurses’, *Nurse Education Today* 35(1), 232–238. https://doi.org/10.1016/j.nedt.2014.09.007

CPD, continuing professional development; EFA, exploratory factor analysis; KMO, Kaiser-Meyer-Olkin; *M*, mean; SD, standard deviation; Career, career opportunities factor; PPD, personal and professional development; Required, requirements factor, which represents the grouping of items obtained by Brekelmans et al. (2015).

#### Confirmatory factor analysis

**FIGURE 2:**
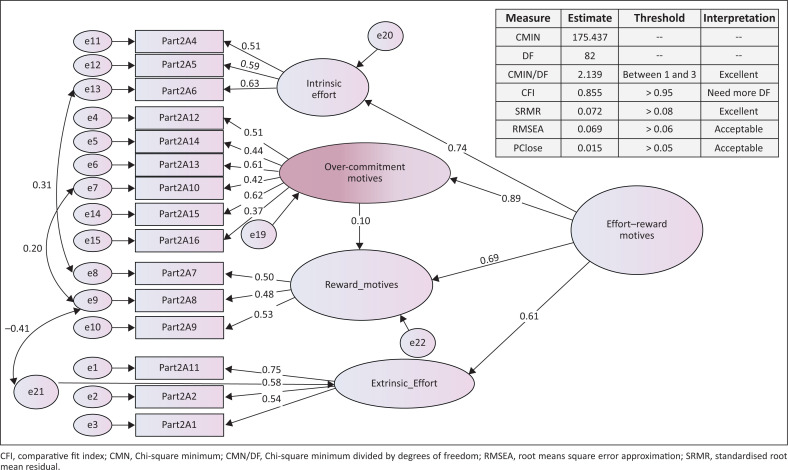
Confirmatory factor analysis model of the effort-reward motives factors.

To conduct CFA, the Rotated Factor Matrix results from the EFA were transferred to SPSS AMOS version 23 using a ‘Pattern Matrix Model Builder’ plugin developed by Gaskin and Lim (2016). The model builder plugin automatically creates a covariance model within SPSS AMOS based on the results of an EFA performed in the conventional SPSS EFA. The researcher employed CFA using the IBM SPSS AMOS 23 software to measure the factor structure and correlations between factors. The challenge in testing the factor structures was in model fitting. As such, the study relied on Gaskin and Lim (2016) developed plugins, which allows one to automate the CFA through iteration of different factor structures as suggested by Gaskin (2017). Whilst the ‘Model fit measures’ (MFM) plugin was used to quickly assess whether the iterated model fitness was good enough. To determine consistency amongst all variables, the MFM plugin prints out the model fitness parameters in a web browser, with suggestions and recommendations on which items are problematic to the model fitness and validity. The plugin output includes the traditional Chi-square value (CMIN), the goodness-of-fit index (GFI), the sample root means square error approximation (RMSEA), comparative fit index (CFI) and PClose fit statistic. The model fitness parameter and thresholds guided by the cut-off criterion from Hu and Bentler (1999) were presented guiding the researcher to the most optimal factor structure. The resultant model fit and its fitness parameters are presented in Figure 2.

### Ethical consideration

Ethical approval was obtained from Stellenbosch University Health Research Ethics Committee nr S16/10/223, and the Ministry of Health and Social Services, Namibia nr 17/3/3.

## Results

Four reflective factors were extracted from the 16 CPD motives using EFA. Siegrist’s ERIERI model was used to label the extracted factors and link them to motives for participation in CPD activities. The four reflective factors included (1) extrinsic efforts, (2) intrinsic efforts, (3) reward motives and (4) over-commitment motives. The four ERI factors were then cross-sectionally aligned to Brekelmans et al.’s (2015) three CPD motives factors (career opportunities, requirements and personal and professional development), which were extracted using the same 16 item Q-PDN instrument. However, there was a mismatch between the grouping of items in this study and that of the items in the study by Brekelmans et al. (2015). Table 1 presents the descriptive and factor analysis results of ERI factors and links these to CPD motives.

Table 1 shows interesting item level results, which highlight significant differences in the reasons nurses in Namibia participate in CPD activities. For instance, reasons such as ‘carry out work better’ (*M* = 3.58, SD = 0.6), ‘make a positive contribution’ (*M* = 3.67, SD = 0.57) and ‘improve quality of work’ (*M* = 3.64, SD = 0.6) achieve higher scores. Reasons such as ‘increased chance of promotion’ (*M* = 2.28, SD = 1.12), ‘improve leadership ability’ (*M* = 2.68, SD = 1.12) and ‘meet requirements for registration in the future’ (*M* = 2.64, SD = 1.19) achieve lower scores.

Given the response anchor scores can range from 1 to 4, a mean of 3.67 is strong agreement. The responses to each item provide detail on which factors are most and least influential in nurses taking part in CPD. Consequently, giving insights into inherent effort–reward factors that can motivate nurses to participate in CPD activities related to the career opportunities, organisational and professional requirements. The item level mean values were also ranked in descending order in order to indicate the most influential factors. The Namibian nurses viewed personal and professional development (PPD) motives ranked at 1–3 as the most influential CPD motive for participating in CPD activities statements. Whilst the extrinsic effort factor was the least influential ERI model factor, as reflected by its indicators such as chances of promotion (*M* = 2.28, SD = 1.12) and registration requirements (*M* = 2.64, SD = 1.19).

Table 1 results also indicated high mean values (above 3) for reward motives (*M* = 3.20–3.58) and intrinsic efforts (*M* = 3.10–3.46). The results also indicated that reward motives were reflected in career opportunities (*M* = 3.53, SD = 0.63), requirements (*M* = 3.2, SD = 0.89) and PPD (*M* = 3.58, SD = 0.60). Additionally, the factors were correlated with demographic variables and significant relationships were observed in the age and education levels of the nurses (Table 2).

**TABLE 2:** Correlational matrix of the effort-reward motives.

Number	Variable	1	2	3	4	5	6
1	Extrinsic effort	1.00†	-	-	-	-	-
2	Intrinsic effort	0.341**	1.00†	-	-	-	-
3	Reward motives	0.187**	0.318**	1.00†	-	-	-
4	Over-commitment motives	0.319**	0.400**	0.389**	1.00†	-	-
5	Age group	−0.08§	−0.183**	0.09‡	0.05‡	1.00†	-
6	Education level	−0.07§	0.00¶	0.10‡	0.184**	0.175**	1.00†

† , Strong positive;

‡ , Moderate positive;

§ , Strong negative;

¶ , Moderate negative.

** , Correlation is significant at the 0.01 level (two-tailed).

Table 2 indicates moderate correlations between factors and small to moderate correlations between the factors and the demographic variables. The findings show the extrinsic effort having a small but significant positive correlation with reward motives (*r* = 0.341, *p* < 0.01), whilst having no significant correlation with age and education. The intrinsic effort factor indicated having significant and moderate positive correlations with all three factors, as well as, having a small but negative correlation with age group (*r* = −0.183, *p* < 0.01). The over-commitment motives factor indicated a small and positive correlation with education level (*r* = 0.175, *p* < 0.01). The findings imply that the older professional nurses in Namibia have lower intrinsic effort motivation than their younger counterparts, whilst the more educated professional nurses indicated had high over-commitment motives.

### The effort–reward motives factor model

The main aim of the article was to propose a model for selected ERI factors, which motivate nurses to participate in continuous professional development activities. The researchers used a CFA measurement model to propose a model for selected ERI factors. The model is presented in Figure 2.

Model fit is assessed using Gaskin and Lim’s criteria (2016). The model fit descriptive measures show that a Chi-square value of 175.437 with 82 degrees of freedom is significant at Chi-square minimum divided by degrees of freedom (CMIN/DF) (17.437/82) value 2.139 (between 1 and 3). These findings suggest that the model fits the data excellently. The RMSEA fit statistic with a value of 0.069 (recommended to be between 0.06 and 0.08) is acceptable. Similarly, the standardised root mean residual (SRMR) of 0.072 (recommended to be < 0.08) is excellent. To solidify, the evidence PClose fit statistic was 0.015 (recommended to be between 0.01 and 0.05). The measures also include the CFI, which was 0.855 (recommended to be > 0.95) compares the absolute fit of the specified model with the absolute fit of the independence model. The model fit descriptive statistics are provided through Hu and Bentler’s (1999) cut-off criteria for fit indexes in covariance structure analysis, which forms the basis of Gaskin and Lim’s (2016) SPSS AMOS plugin for model fit measures.

Based on the coefficients and correlations, the analysis of construct reliability, convergent and divergent validity was conducted. The findings in Table 3 indicate that the factors failed to meet the criteria for construct reliability and convergent validity but met the criteria for divergent validity. Whilst the model fit appears to be good, there is a room for improvement in the measurement of the motivation factors. Therefore, future research using the scale would need to revise the wording of statements within factors and strengthen the operationalisation of intrinsic and extrinsic effort, over-commitment and reward factors.

**TABLE 3:** Validity and reliability of the factors.

Measure	Factors	Intrinsic	Over-commitment	Reward	Extrinsic
**Composite reliability**					
Measure	Joreskog rho	0.6	0.663	0.505	0.659
Condition	Is J rho > 0.7?	N	N	N	N
**Convergent validity**					
Measure	AVE	0.335	0.254	0.254	0.397
Condition	Is AVE > 0.5?	N	N	N	N
**Discriminant validity**					
Measure	MSV	0.16	0.16	0.151	0.116
Condition	Is MSV < AVE?	Y	Y	Y	Y
Measure	ASV				
Condition	Is ASV < AVE?	Y	Y	Y	Y
Measure	SQRT(AVE)				
Condition	Is SQRT(AVE) > interconstruct correlations?	Y	Y	Y	Y
Do not meet the construct reliability and convergent validity criteria but discriminant validity is acceptable	-	-	-	-	-

AVE, average variance extracted; MSV, maximum shared variance; ASV, average shared variance; SQRT, square root of AVE.

## Discussion

This article measured the effort and reward factors, which motivated nurses to participate in CPD activities in order to avoid the types of imbalances, as described by Kluska et al. (2004). The effort–reward motivation had four factors, which were extracted from 16 items measuring reasons and motivations for nurses’ participation in professional development activities. These factors were as follows: reward motives, intrinsic effort, extrinsic effort and over-commitment motives. The three factors, reward motives, intrinsic effort and over-commitment motives were rated high whereas the extrinsic effort factors were rated low by the professional nurses.

### Reward motives

In line with previous findings (Noor & Zainordin 2018), the reward motives factor was rated with high means; this could mean that professional nurses in Namibia are motivated to take part in CPD activities because of an interest in supporting their nursing career and also to ensure that the organisational requirements are met. Furthermore, this study found that professional nurses indicated and agreed that they were motivated to take part in CPD activities in order to carry out their work better. Indeed, Beaudoin, Alderson and St-Louis (2014) found and indicated that taking part in professional growth and development activities help professional nurses to improve their confidence about their communication skills with regard to patient care and management and this will enable them to perform their work better. Similarly, Sykes and Temple (2012) maintained that patients directly benefit from professional nurses who are lifelong learners and those who recognise the need to update their knowledge because it can be a rewarding factor at personal and professional levels. Thus, based on previous research, it can be concluded that reward motives play an important role in building and sustaining employees’ commitments, thus ensuring high standards of performance and consistency in the workforce.

### Intrinsic effort

The findings from the aspects in this factor indicated that professional nurses in Namibia agreed that they were motivated to take part in CPD activities because it was considered important to increase the status of the profession. The high rating of this factor supports the notion that professional nurses are required to decide and indicate what naturally motivates them to participate in CPD activities. Furthermore, the intrinsic effort factors require nurses to indicate whether their participation in CPD enables them to improve their qualifications. Indeed, in the patient charter of Namibia, patients expect to be treated by professional nurses who are skilful, knowledgeable and competent, and that nursing care should take place in a safe environment (Ministry of Health and Social Services 2016). Even more important, professional nurses also agreed that they take part in CPD activities to improve their current qualifications. McNeely et al. (2015) found similar results, which demonstrated that the progressive effects resulting from additional qualifications include increased productivity, few work-related injuries and staff retention. Similarly, intrinsic factors such as job security, responsibility and achievement were also found to significantly and positively influence employees.

### Over-commitment motives

Professional nurses indicated that they would be motivated to over-commit and take part in CPD activities because it is considered highly important in their professional development. This is in keeping with the literature Pool et al. (2015) found in their study that professional development strategies assist nurses to keep up to date with latest knowledge and skills. Indeed, over-commitment was found to be an intellectual and a motivational arrangement, which is characterised by being excessively involved in the job. In addition, professional development assists professional nurses to perform other roles within the work environments (Badu-Nyarko 2015). Thus, the professional nurses in this study perceived the work environment as welcoming, supportive and providing opportunities to participate in CPD activities. Equally important, professional nurses in this study indicated that they were motivated to over-commit and take part in CPD activities in order to make positive contribution to nursing practice. This result was comparable with the previous study (Satoh, Watanabe & Asakura 2017), which found that occupational commitment is crucial for influencing nurses’ work attitudes and outcomes which are issues important for professional development.

### Extrinsic effort

The low rating of this factor was expected because in Namibia, participation in continuous professional development activities does not increase the chances of promotion, nor does it serve as a requirement for future registration with the nursing council. However, previous research indicates that determinants such as promotion, financial and non-financial rewards are some of the extrinsic motivation determinants, which externally motivate nurses to perform their duties (Akpasubi & Callum 2018). Similarly, employees who were found to be extrinsically motivated tend to have low problem-solving skills, low levels of creativity and high levels of money orientation. Indeed, it was evident that professional nurses who participated in this study were not extrinsically motivated to participate in continuous professional development activities because of the determinants related to the extrinsic effort rewards. On the contrary, wages, benefits and satisfaction were found to be important extrinsic job characteristics reflected in job satisfaction, which is shaped by the external work environment (Sell & Cleal 2011).

### Limitations

This study was conducted in a public national referral hospital where conditions were not the same as in other hospitals in Namibia and elsewhere in the world. Therefore, this could limit generalisation of the research findings to professional nurses working in other healthcare facilities in Namibia and elsewhere in the world. Furthermore, although the study used a previously validated data collection instrument, it was used for a different setting and the authors of the original instrument measured a different dependent variable.

## Conclusion

The study findings indicate that professional nurses rated the ERI factors as strongly influencing their participation in continuous professional development activities. It is thus concluded that the skills and expertise of professional nurses should be recognised and incentivised to assist in reducing staff turnover and job dissatisfaction. It was evident from the results of this study that professional nurses consider taking part in continuous professional development activities in order to carry out their work better but not as a way to increase chances of promotion. In addition, professional nurses are also motivated to participate in continuous professional development activities because they want to make positive contributions to the entire nursing practice. Therefore, it is recommended that healthcare institutions must consider developing systems of rewarding nurses who have demonstrated their interest in continuing education through acquisition of postgraduate qualifications. In addition, long-serving nurses also need to be considered for the reward system. Reward systems could be financial or non-financial systems, such as certificates of recognition, cash bonuses and/or experience-related payments.
