# Erratum: Effort and reward imbalance factors motivating Namibian professional nurses to participate in continuous professional development: A confirmatory factor analysis

**DOI:** 10.4102/hsag.v26i0.1762

**Published:** 2021-11-02

**Authors:** Tekla S.N. Mbidi, Anneleen Damons

**Affiliations:** 1 Department of Nursing and Midwifery, Faculty of Medicine and Health Sciences, Stellenbosch University, Cape Town, South Africa; 2 International University of Management, Windhoek, Namibia

In the version of this article initially published, Mbidi, T.S.N. & Damons, A., 2020, ‘Effort and reward imbalance factors motivating Namibian professional nurses to participate in continuous professional development: A confirmatory factor analysis’, *Health SA Gesondheid* 25(0), a1313. https://doi.org/10.4102/hsag.v25i0.1313, the second author’s affiliation was given incorrectly in the ‘Affiliation’ section. The correct affiliation should be ‘Department of Nursing and Midwifery, Faculty of Medicine and Health Sciences, Stellenbosch University, Cape Town, South Africa’ instead of ‘International University of Management, Windhoek, Namibia’.

This correction does not alter the study’s findings of significance or overall interpretation of the study’s results. The publisher apologises for any inconvenience caused.

